# Improved liver lipid catabolism and utilization in *growth hormone* transgenic common carp (*Cyprinus carpio* L.) through enhanced lipolytic and fatty acid β-oxidation pathways

**DOI:** 10.3389/fendo.2022.982488

**Published:** 2022-09-12

**Authors:** Yunya Wu, Rui Li, Xingxing Wu, Wei Guo, Yongming Li, Yanlong Song, Binbin Tao, Ji Chen, Dong Han, Shouqi Xie, Yaping Wang, Zuoyan Zhu, Wei Hu

**Affiliations:** ^1^ State Key Laboratory of Freshwater Ecology and Biotechnology, Institute of Hydrobiology, Innovation Academy for Seed Design, Chinese Academy of Sciences, Hubei Hongshan Laboratory, Wuhan, China; ^2^ College of Advanced Agricultural Sciences, University of Chinese Academy of Sciences, Beijing, China; ^3^ School of Basic Medical Science, Wuhan University, Wuhan, China; ^4^ Qingdao National Laboratory for Marine Science and Technology, Qingdao, China

**Keywords:** *growth hormone* transgene, common carp (*Cyprinus carpio* L.), lipid catabolism, lipid utilization, fat deposition

## Abstract

*Growth hormone* (*GH*) transgenic common carp (*Cyprinus carpio* L.) show desirable aquaculture traits. Their specific growth rate (SGR) and feed efficiency (FE) are approximately 12% and 17% higher than the wild-type (WT) common carp, respectively. However, the mechanisms of lipid catabolism (lipolysis and fatty acid β-oxidation) and utilization in *GH* transgenic common carp are still unclear. In this study, we firstly compared the lipid metabolism of *GH* transgenic (initial weight 3.72 ± 0.32 g) and WT (initial weight 3.30 ± 0.28 g) common carp fed with a normal fat level diet (6% lipid, 33% protein) for two months, then compared the growth performance of *GH* transgenic (initial weight 3.65 ± 0.33 g) and WT (initial weight 3.27 ± 0.26 g) common carp fed with different fat levels diets (6% lipid and 12% lipid, 33% protein) for two months. We found that the lipid content in serum, liver and whole body was significantly reduced in *GH* transgenic common carp, the hepatic activities of the lipolytic enzymes hormone-sensitive lipase and adipose triglyceride lipase were enhanced, and the hepatic expression level of *hormone-sensitive lipase* was upregulated. In addition, the mitochondrion numbers were increased, and the expression level of *carnitine palmitoyltransferase-1a* and *carnitine palmitoyltransferase-1b* was upregulated in the liver of *GH* transgenic common carp. *GH* transgenic common carp showed higher weight gain and SGR than that in WT carp when fed with a normal-fat diet as they did when fed with a high-fat diet, and *GH* transgenic common carp showed higher FE than that in WT carp when fed with a high-fat diet. These results suggested that the lipid catabolism and utilization was improved in the *GH* transgenic common carp liver through enhanced lipolytic and fatty acid β-oxidation pathways. Our study provides new insights into improving lipid utilization in some aquaculture fish species.

## Introduction

Transgenic technology can directionally modify the genetic characteristics of agricultural organism to improve their economic traits (1–3). Since the first application of transgenic technology to fish genetic modification in the 1980s, a variety of transgenic fish have been developed with improved traits, including faster growth, stronger stress resistance, and higher level of omega3 fatty acid in body (4). Among the exogenous genes chosen to be transplanted, the *growth hormone* gene is applied the most times in fish genetic improvement research, and several *GH* transgenic fish lines that can transmit transplanted *GH* to their offspring stably have been generated, including *GH* transgenic Atlantic salmon (*Salmo salar*) (5), *GH* transgenic tilapia (*Oreochromis* sp.) (6), *GH* transgenic mud loach (*Misgurnus mizolepis*) (7), *GH* transgenic coho salmon (*Oncorhynchus kisutch*) (8) and *GH* transgenic common carp (*Cyprinus carpio* L.) (9, 10), all of which exhibited excellent breeding traits such as higher growth rate and higher feed conversion (8, 11–14). It is also worth mentioning that the *GH* transgenic Atlantic salmon was approved by the US Food and Drug Administration as the first edible genetically engineered animal in 2015 (15), which was recorded as a historic breakthrough in the research and application of transgenic animal breeding worldwide

Before the industrialized culture of *GH* transgenic fish, it is critical to study their metabolic characteristics and nutritional requirements so as to develop the befitting feed to cater to the fast growth demands. Among nutrients required by fish such as protein, lipid, carbohydrate, minerals, micronutrient and vitamins, protein is the most expensive raw material component in aquafeeds. Compared with protein, lipid is a cheaper source of energy for aquaculture fish and is one of the key nutrients that can affect growth performance (16–18). Therefore, some researchers have suggested a certain increasing of the lipid content in the feed to reduce the use of dietary protein (19), achieving a protein sparing effect to save costs (20). However, excess lipid in the feed might affect fish intestinal health (21), inhibit growth (22), and cause lipid accumulation in the liver of fish, resulting in fatty liver (23).

In fish, GH is secreted from the pituitary gland, which binds to GH receptors distributed in organs including the liver through circulation, and promotes growth *via* Insulin-like growth factor-1 (24). The liver is one of the main organs involved in energy metabolism and is responsible for the synthesis and decomposition of protein, lipid, and carbohydrate. A study in mammals has found that GH promoted lipolysis by enhancing the activity of hormone-sensitive lipase (HSL) in visceral adipose tissue (25). Studies in *GH* transgenic coho salmon and Atlantic salmon have found that consistently expressed *GH* increased the expression level of hepatic lipid synthesis-related genes (26, 27). Other studies in *GH* transgenic coho salmon also found continuous expression of *GH* enhanced lipid synthase activity in the liver (28) and increased whole body lipid content (29). It has also been reported that whole body lipid content of *GH* transgenic coho salmon increased with dietary energy level (30). Studies in *GH* transgenic common carp found that continuous expression of *GH* led to an increase in the expression level of lipolysis-related genes (31), a decrease in the expression level of hepatic lipid synthesis-related genes (32, 33), and a decrease in whole body lipid content (10). Various changes in hepatic lipid metabolism caused by overexpressed *GH* in different fish species might suggest the complexity in mechanism of GH action. For *GH* transgenic fish, how exactly continuous expression of GH can promote growth by affecting lipid metabolism is still unclear.

For the aim of evaluating lipid utilization ability of *GH* transgenic common carp, as well as the effect of dietary lipid level on growth performance, we performed this study. By comparing the differences in growth performance, lipid accumulation, lipid metabolism-related enzyme activities, and gene expression level between the *GH* transgenic and the wild-type (WT) common carp, together with liver transcriptome analysis, we found that *GH* overexpression primarily enhanced the capacity for lipid catabolism in the liver of the common carp. Common carp maintained the same growth performance by intaking more feed when fed with a high-fat diet. Correspondingly, *GH* transgenic common carp had a higher lipid utilization rate than WT carp when fed with a high-fat diet.

## Materials and methods

### Expt. 1: Lipid metabolism fed with a normal-fat diet

#### Experimental fish and diets

*GH* transgenic fish fry in this study were the offspring of the *GH* transgenic common carp family which inherited *GH* transgene stably (10), with higher serum GH level (34). The fry of the *GH* transgenic and WT common carp hatched on the same day were fed with commercial feed (6% lipid, 33% protein, Tongwei, China) twice a day until 2 months of age at the Liangzi Lake Breeding Base of the Institute of Hydrobiology, Chinese Academy of Sciences (Wuhan, China). From 2 weeks before the trial, all fish were fed with a normal-fat diet (NF, 6% lipid, 33% protein, **Supplementary Table 1**) twice daily (9:00, 17:00) to make them adapt to the circulating water system to ensure they can intake and grow normally during the feeding experiment. At the beginning of the trial, all fish were fasted for 24 h. Healthy (no damage on the surface of the fish, no disease, and normal swimming behavior) and similar-sized *GH* transgenic (initial weight 3.72 ± 0.32 g, 2 months old) and WT (initial weight 3.30 ± 0.28 g, 2 months old) common carp were weighted and distributed into 3 tanks (diameter: 80 cm; water volume: 400 L) with 30 fish each tank in a recirculating aquaculture system, respectively. Each fish strain was randomly assigned into three tanks. Fish were fed with a NF diet (6% lipid, 33% protein) to satiation twice a day (9:00, 17:00) for 8 weeks. During the experiment, the water temperature ranged from 30.1°C to 31.1°C, dissolved oxygen content was above 6.0 mg/L, ammonia nitrogen content was less than 0.4 mg/L, and photoperiod was 12 h light:12 h dark with a light period from 7:00 to 19:00.

#### Fish sampling

At the end of the 8-week feeding trial, all fish in each tank were fasted overnight, and weighed for determination of final body weight. Final body weight of *GH* transgenic and WT common carp were 78.44 ± 0.49 g and 53.60 ± 0.70g, respectively. Six fish from *GH* transgenic and WT common carp each (two fish per tank) were randomly chosen and euthanized with 40 mg/L Ethyl 3-aminobenzoate methanesulfonate (Macklin, China) for serum collection. The blood was drawn from caudal vein using 2 mL syringes, then centrifuged at 4°C by 1,500 rpm for 30 min. The serum was isolated and stored at −80°C for later biochemical analysis. Next, six individuals from *GH* transgenic and WT common carp each were dissected for collecting liver samples. To avoid repeated freezing and thawing, each liver sample was divided into five pieces in separate tubes, instantly frozen in liquid nitrogen, then stored at −80°C for later RNA isolation, biochemical analysis, and histological analysis. Another nine individuals from *GH* transgenic and WT common carp each (three fish per tank) were sacrificed as described above and stored at −20°C later for body proximate composition analysis (n=6, two fish per tank) and micro-CT (n=3, one fish per tank).

#### Fasting experiment

At the end of the 8-week feeding trial, 50 individuals from *GH* transgenic with uniform size at 4 months of age and 50 individuals from WT common carp with uniform size at 4 months of age were fasted for 6 days. During the fasting period, the sampling time points were 4 h, 12 h, 1 d, 2 d, 4 d, and 6 d. Four liver samples from *GH* transgenic and WT common carp each were collected and frozen at −80°C for later RNA isolation and biochemical analysis at every time point. Another four fish from *GH* transgenic and WT common carp each were sacrificed as described above and stored at −20°C for later body proximate composition analysis at 1 d – 6 d.

#### The proximate composition analysis

The content of crude lipid, crude protein, ash and moisture of the experimental diets were determined using standard methods (35). The crude lipid content was determined by Soxhlet extraction using a Soxhlet extractor (Soxtec-2055, FOSS, USA). The crude protein content was determined using the Kjeldahl method (Kjeltec Auto Analyzer 4800, FOSS, USA). The moisture content was determined by drying the sample to a constant weight at 105°C. The ash content was determined by combustion in muffle furnace at 550°C. The gross energy was calculated as described by Tan et al. (36), and the formula is as the following: Gross energy (KJ/g dry matter) = (% crude protein × 23.62 + % lipid × 38.87 + % carbohydrate × 17.15)/100, Carbohydrate=1 − (% crude protein + % total lipid + % ash).

The crude protein content of four fish samples from fasting experiment and the crude lipid content of three fish samples from feeding trial were determined as described above. The total lipid of six liver samples from feeding trial was extracted and determined by using chloroform/methanol (2:1, *v*:*v*) method (37). Three independent duplicates were conducted for each of the data.

#### Biochemical parameters measurement

We assessed biochemical parameters by using commercial assay kits (Nanjing Jiancheng, China) according to the manufacturer’s protocols. The absorbance of dye for all biochemical parameters was measured on a microplate reader (FlexStation 3, Molecular Devices, USA). The contents of glycogen and triglyceride in the liver from fasting experiment were determined using Anthranone method (Absorbance at 620 nm) and GPO-PAP method (Absorbance at 510 nm), respectively. The concentrations of high-density lipoprotein cholesterol (HDL-C) and low-density lipoprotein cholesterol (LDL-C) in the serum were determined using Colorimetric method (Absorbance at 546 nm) and the concentration of apolipoprotein B (ApoB) was determined using Immunoturbidimetry method (Absorbance at 340nm) from feeding trial. Similarly, the content of triglyceride, non-esterified fatty acid (NEFA) and total cholesterol (T-CHO) in the serum and liver from feeding trial was determined using GPO-PAP method (Absorbance at 510 nm), ACS-PAP Method (Absorbance at 546 nm) and COD-PAP Method (Absorbance at 510 nm), respectively. The activities of fatty acid synthase (FAS), acetyl-CoA carboxylase (ACC), adipose triglyceride lipase (ATGL), and hormone-sensitive lipase (HSL) in the liver from feeding trial were measured by using enzyme-linked immunosorbent assay (ELISA) reaction (Absorbance at 450nm). Three independent duplicates were conducted for each of the data.

#### Morphology and histology analysis

Six liver samples from *GH* transgenic and WT common carp each were fixed in 4% PFA at 4°C overnight. After dehydration with 30% sucrose, the samples were embedded in O.C.T. compound and cryosectioned. As described previously (38), the frozen sections were stained with Oil Red O to visualize fat deposits in the liver. The images were observed and captured by a microscope with a CCD (Aperio Versa 8 Scanner, Leica, Germany), then the relative area of lipid droplet was analyzed using Image-Pro Plus 6.0. A number of 4-6 pictures were taken per fish.

To measure the volume of total adipocyte tissue in common carp, the whole body of three fish were dried using blotting paper, and then scanned with a micro-CT instrument (Skyscan 1276, Bruker, Belgium) using the following settings: source voltage, 85 kV; source current, 200 µA; AI 1 mm filter; pixel size 40 µm; rotation step, 0.5 degree. The images were then reconstructed with NRecon software (Bruker, Belgium).

#### Real-time quantitative polymerase chain reaction (qPCR) analysis

sTotal RNA was exacted from the liver from feeding trial and fasting experiment using TRIzol reagent (Invitrogen, USA) according to the manufacturer’s protocol. After DNase I (Promega, USA) treatment, 1 μg total RNA was used for the reverse transcription using a ReverTra Ace M-MLV kit (TOYOBO, Japan). The cDNA sample was diluted 10 times with RNase-free water, and qPCR was performed on a Bio-Rad CFX384 Real-Time PCR System (Bio-Rad, USA). The reaction system consisted of 5 µL 2x SYBR Green Realtime PCR Master Mix (Toyobo, Japan), 1 µL (5 µM) of forward primer, 1 µL (5 µM) of reverse primer, 1 µL H_2_O, and 2 µL cDNA. The qPCR was performed at 95°C for 2 min, followed by 40 cycles of 95°C, 15 sec; 60°C, 20 sec; 72°C, 30 sec. The primers’ sequences were listed in **Supplementary Table 2**. The relative mRNA expression was calculated using 2^−ΔΔCt^ method, and the *β-actin* gene was used as a reference gene as previously described (39). Three independent duplicates were conducted for each of the data.

Mitochondrial numbers were assessed by determining the quantity of mitochondrial *cytochrome b* (*cytb*) DNA, as previously described (40). Using liver DNA as a template and *β-actin* as a reference gene, the relative copy number of mitochondrial DNA (mtDNA) was measured *via* qPCR using mitochondrial *cytb*/*β-actin*. The qPCR reaction condition was the same as described above, and the primers’ sequences are listed in **Supplementary Table 2**. Three independent duplicates were conducted for each of the data.

### Expt. 2: Liver transcriptome analysis

#### RNA isolation, library construction, sequencing, and data analysis

The 6-month-old *GH* transgenic (761.02g ± 33.17g) and WT common carp (533.53g ± 10.44g) were transferred from the Breeding Base to the indoor constant-temperature circulating water system and were fed with commercial diets for 8 weeks.

Three liver samples from *GH* transgenic and WT common carp, respectively, were collected. Total RNA was isolated using TRIzol reagent (Invitrogen, USA), then treated with DNase I (Promega, USA). The purity, quantity, and integrity of the extracted total RNA were measured using a NanoDropND-2000 spectrophotometer (Thermo, USA), 1.2% (w/v) agarose gel electrophoresis, and an Agilent 2100 Bioanalyzer (Agilent Technologies, Richardson, USA), respectively. RNA with an RNA Integrity Number (RIN) > 8, 28S/18S > 0.7, and A260/280 values of approximately 2.0 was used to construct the RNA-seq library. Poly (A) mRNA was isolated from the total RNA using poly (dT) oligo-attached magnetic beads, and cDNA libraries were prepared using the TruSeq RNA Sample Preparation Kit (Illumina, USA), then sequenced on the Illumina Hiseq X Ten platform. Clean data were obtained by removing redundant reads, and then mapped to the common carp reference genome (41). Differential expressed gene (DEG) analysis was performed using the Deseq2 algorithm (42). According to the annotation results of Kyoto Encyclopedia of Genes and Genomes (KEGG, https://www.kegg.jp/) and the official classification, we classified the DEGs into biological pathways, and used the phyper function in the R software for enrichment analysis.

#### Validation of DEGs by qPCR

In order to confirm the reliability of the data obtained by RNA-seq, two DEGs from synthesis and degradation of ketone bodies pathway, and two others from fatty acid degradation pathway, which we concerned, were selected for validation using qPCR. The primers were listed in **Supplementary Table 3**. The qPCR reaction condition was the same as described in Expt 1. Three replicates were conducted for each sample, and *β-actin* gene was used as an internal control. Relative expression level was calculated using the 2^−ΔΔCt^ method.

### Expt. 3 Growth experiment fed with different fat level diets

The NF and high-fat (HF) diets (**Supplementary Table 1**) were prepared. Since an optimal fat content in diet for common carp is approximately 5% ~ 8% (43–45), the 6% fat content diet was designed as NF diet and the 12% fat content diet was designed as HF diet. White fish meal, soybean meal and casein were the major protein sources, corn starch was the carbohydrate source, and fish oil and soybean oil were the major fat sources. The lipid content in the NF diet and HF diet is 6% and 12%, respectively. All the ingredients were passed through a 40-mesh sieve and mixed. The wet dough was extruded into 2 mm pellets using a laboratory extruder (Mechanical Facility Research Institute, China). The pellets were dried at 70°C and then stored at 4°C.

Before the trial, all fish were fed with an equal mixture of experimental diets twice daily (9:00, 17:00) for 2 weeks to acclimatize them to the experimental conditions. Before the trial, all fish were fasted for 24 h. Healthy and similar-sized *GH* transgenic (initial weight 3.65 ± 0.33 g, 2 months old) and WT (initial weight 3.27 ± 0.26 g, 2 months old) common carp were batch-weighted and distributed into 6 tanks (diameter: 80 cm; water volume: 400 L) with 30 fish each tank in a recirculating aquaculture system, respectively. Each experimental diet-fish strain was randomly assigned to three tanks. Fish were fed to satiation twice a day (9:00, 17:00) for 8 weeks. During the experiment, the culture environment was the same as described in Expt. 1.

After 8 weeks, the fish in each group were weighted to calculate the weight gain rate (WG), specific growth rate (SGR), and feed efficiency (FE) using the following formulas:


WG=100×[final body weight (g)−initial body weight (g)]/initial body weight (g)

SGR=100×{Ln[final body weight (g)]−Ln[initial body weight (g)]}/d

FE=100×[final body weight (g)−initial body weight (g)]/dry feed fed (g)

### Statistical analysis

All data were presented as the mean + standard error of mean (SEM). Statistical differences in the measured variables between two groups were tested by Student’s unpaired t-test using GraphPad Prism 8. Statistical comparisons for measured parameters containing more than two groups were performed using one-way analysis of variance (ANOVA) followed by least significant difference (LSD) test for specific comparisons among groups whenever differences were obtained using SPSS Statistics 22. Data regarding final body weight, WG, SGR, feed intake and FE were to estimate the significant difference between different strains under one diet condition or between NF and HF condition for one strain. These data were further analyzed by two-way ANOVA to determine the interaction between *GH* transgene and dietary lipid contents. Statistical significance was set at *P*< 0.05.

## Results

### Utilization of energy source during fasting

**Figure 1:**
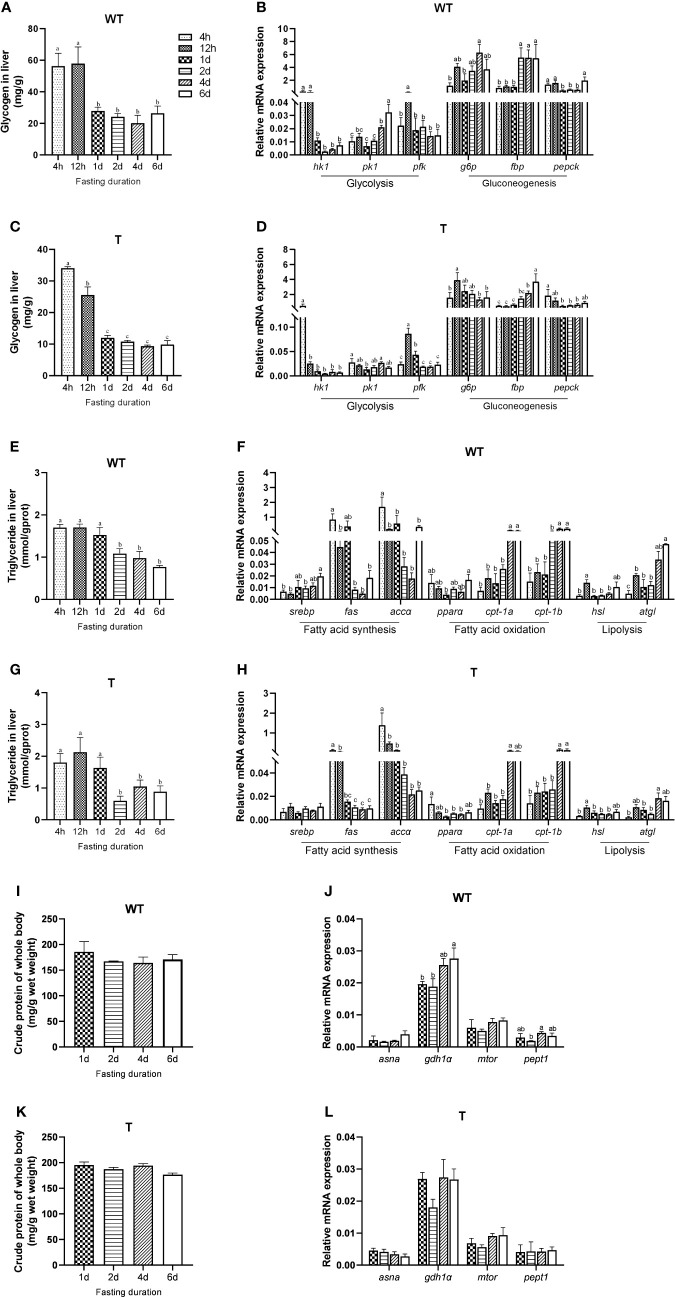
Mobilization of different energy sources during fasting of wild-type (WT) and GH transgenic **(T)** common carp (n = 4). **(A, C)** Hepatic glycogen content during fasting in WT and T common carp. **(B, D)** Hepatic expression level of genes involved in glycolysis and gluconeogenesis during fasting in WT and T common carp. **(E, G)** Hepatic triglyceride content during fasting in WT and T common carp. **(F, H)** Hepatic expression level of genes involved in lipogenesis and lipid catabolism during fasting in WT and T common carp. **(I, K)** Protein content of whole body during fasting in WT and T common carp. **(J, L)** Hepatic expression level of genes involved in protein catabolism during fasting in WT and T common carp. Statistical significance was determined *via* one-way analysis of variance. Different letters show significant differences among groups (*P*< 0.05).

Different fish species had different strategies in utilizing their energy reserves. For *GH* transgenic and WT common carp, the order of utilizing glycogen, lipid, and protein during short-term fasting has not yet been reported. Therefore, we firstly examined the mobilization of carbohydrate, lipid and protein of *GH* transgenic and WT common carp under short-term starvation. Similar to that in the WT common carp, the glycogen content and the expression level of glycolysis-related genes (*phosphofructokinase*, *pfk*) in the liver of the *GH* transgenic common carp markedly decreased after 12 hours of starvation, and liver glycogen content remained at a lower level compared to 4h-12h of starvation during subsequent starvation (**Figures 1A–D**). The triglyceride content in the liver of WT common carp significantly decreased after one day of starvation (**Figure 1E**), and the expression level of lipid synthesis-related genes (*fatty acid synthase*, *fas*; *acetyl-CoA carboxylase α*, *accα*) in the liver of WT common carp decreased although not significantly after one day of starvation, and the expression level of fatty acid β-oxidation-related genes (*carnitine palmitoyltransferase-1a*, *cpt-1a*; *carnitine palmitoyltransferase-1b*, *cpt-1b*) and lipolysis-related genes (*adipose triglyceride lipase*, *atgl*) increased significantly in the late starvation period (4 d – 6 d) (**Figure 1F**). The changing trend of liver triglyceride content and lipid metabolism-related gene expression level in *GH* transgenic common carp was consistent with that of WT common carp (**Figures 1G, H**). The protein content of the *GH* transgenic and WT common carp did not change during the six days of starvation. However, the expression level of such genes as *glutamate dehydrogenase 1α* (*gdh1α*), *oligopeptide transporter 1* (*pept1*) in the liver of WT common carp changed during starvation, and the expression level of other protein metabolism-related genes did not change during starvation (**Figure 1I–L** and **Supplementary Table 4**). These data showed that *GH* transgenic common carp mainly mobilized carbohydrate at first for energy supply in the early stage of starvation (within 1 d), and lipid was mostly utilized after glucose catabolism, while protein catabolism was not activated during short-term starvation (6 d), so did WT common carp.

### Effects of *GH* overexpression on lipid accumulation, enzymatic activities and expression of genes related to lipid metabolism

**Figure 2:**
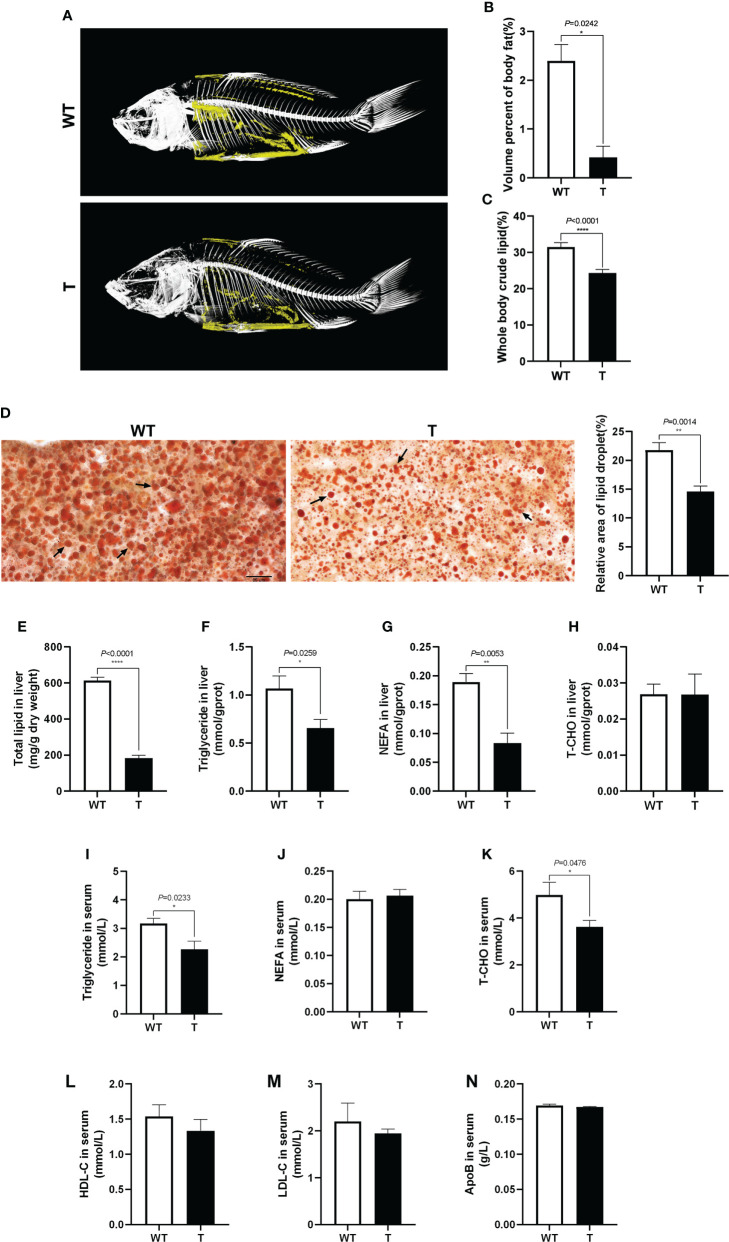
Effects of GH overexpression on lipid accumulation in common carp. **(A)** Mirco-CT of wild-type (WT) and GH transgenic **(T)** common carp (n = 3). The body fat in the fish was colored yellow. **(B)** The statistical graph of body fat by micro-CT of WT and T common carp (n = 3). **(C)** Whole body crude lipid of WT and T common carp (n = 3). **(D)** The liver slices were stained with Oil Red O. Scale bars, 25 μm (40×) (n = 6), the lipid droplet was indicated by arrows, the column graphs in the right of pictures represent the relative lipid droplet area of liver tissue. **(E–H)** Total lipid, triglyceride, NEFA, and T-CHO content in liver of WT and T common carp (n = 6). **(I–K)** Triglyceride, NEFA, and total cholesterol (T-CHO) content in serum of WT and T common carp (n = 6). **(L–N)** High-density lipoprotein cholesterol (HDL-C), low-density lipoprotein cholesterol (LDL-C), and apolipoprotein B (ApoB) content in serum of WT and T common carp (n = 6). *, **, and **** indicate 0.01 < *P*< 0.05, 0.001 < *P*< 0.01, *P*< 0.0001, respectively, between WT and T common carp.

To clearly show the lipid accumulation in *GH* transgenic and WT common carp, the fish were scanned with micro-CT. As shown in **Figures 2A, B**, the body fat content of *GH* transgenic common carp was significantly lower than that of the WT common carp (*P*< 0.05), which was consistent with the crude lipid content of the fish measured by diethyl ether extraction (**Figure 2C**). The liver tissue was stained with Oil Red O, and it was found that the the relative area of lipid droplet in the liver of *GH* transgenic common carp was significantly lower than that in WT common carp (*P*< 0.05) (**Figure 2D**), which was consistent with the total lipid content in the liver measured by chloroform-methanol extraction and the triglycerides content in the liver measured by commercial kit using GPO-PAP method (Absorbance at 510 nm) (**Figures 2E, F**). The content of NEFA in the liver of *GH* transgenic common carp was 55.82% lower than that in WT common carp (**Figure 2G**). The content of T-CHO in the liver of *GH* transgenic and WT common carp did not differ significantly (*P* > 0.05) (**Figure 2H**). In addition, the triglyceride and T-CHO contents in the serum of *GH* transgenic common carp were 28.60% and 27.24% lower than those of WT common carp, respectively (**Figures 2I, K**). There were no significant differences in the contents of NEFA and apolipoproteins (HDL-C, LDL-C and ApoB) in the serum between *GH* transgenic and WT common carp (*P* > 0.05) (**Figures 2J, L–N**). These results indicated that the lipid content in the serum, liver and whole body of *GH* transgenic common carp decreased.

**Figure 3:**
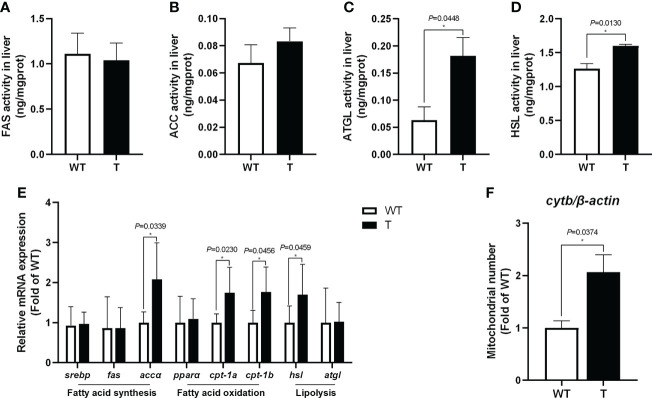
Effects of GH overexpression on enzymatic activities and relative expression level of genes related to lipid metabolism in common carp. **(A–D)** Enzymatic activities of fatty acid synthase (FAS), acetyl-CoA carboxylase (ACC), adipose triglyceride lipase (ATGL), and hormone-sensitive lipase (HSL) in the liver of wild-type (WT) and GH transgenic **(T)** common carp (n = 6). **(E)** Relative expression level of genes involved in lipid metabolism in the liver of WT and T common carp (n = 6). **(F)** Mitochondrial copy number in the liver of WT and T common carp (n = 6). * indicates 0.01<*P*<0.05 between WT and T common carp.

Compared with that in WT common carp, the activities of lipolytic enzymes (ATGL and HSL) in the liver of *GH* transgenic common carp were significantly enhanced (*P*< 0.05) (**Figures 3C, D**). Moreover, the expression level of genes related to lipolysis, such as *hsl*, in the liver of *GH* transgenic common carp was significantly higher than that in WT common carp (**Figure 3E**). The activities of lipid synthase (FAS and ACC) in the liver of *GH* transgenic and WT common carp did not differ significantly (*P* > 0.05) (**Figures 3A, B**). In addition, the quantity of mtDNA of *cytb*, a marker for mitochondrion number, increased significantly in the liver of *GH* transgenic common carp (**Figure 3F**). Additionally, the expression level of genes related to fatty acid β-oxidation, such as *cpt-1a* and *cpt-1b*, in the liver of *GH* transgenic common carp were significantly higher than those in WT common carp (**Figure 3E**).

### Transcriptome analysis of the liver of the *GH* transgenic and WT common carp

**Figure 4:**
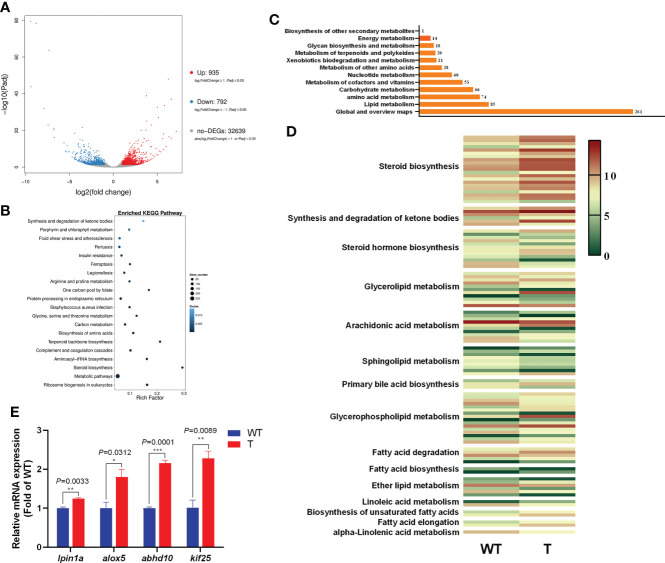
Transcriptome analysis of the differentially expressed genes (DEGs) in the liver of wild-type (WT) and GH transgenic **(T)** common carp. **(A)** Volcano-plot map of DEGs. **(B)** Enriched KEGG pathway of the DEGs in the liver of WT and T common carp; the size of the point represents the number of DEGs (the larger the point, the larger the number). **(C)** Functional classification of DEGs in metabolic pathways. **(D)** Heat map of the expression level of genes involved in lipid metabolism; red and green indicate higher and lower expression, respectively. **(E)** qPCR validation of DEGs. *, ** and *** indicate 0.01<P<0.05, 0.001<P<0.01, 0.0001<P<0.001, respectively, between WT and T common carp.

To examine the effect of *GH* overexpression on the metabolism in the liver of common carp, we performed transcriptome analysis on the liver of 8-month-old *GH* transgenic and WT common carp. We identified 1,727 DEGs in the liver between *GH* transgenic and WT common carp, of which the expression level of 935 genes was upregulated, and of which the expression level of 792 genes was downregulated in the *GH* transgenic fish (**Figure 4A**). Based on differential expression analysis, we conducted KEGG biological pathway classification and enrichment analysis, and most DEGs were found to be enriched in metabolic pathways (**Figure 4B**). Among the metabolic pathways, the most enriched pathway was lipid metabolism (**Figure 4C** and **Supplementary Table 6**-**8**). In lipid metabolism, the DEGs with upregulated expression level in the liver of *GH* transgenic common carp were primarily enriched in pathways such as steroid biosynthesis, synthesis and degradation of ketone bodies, glycerolipid metabolism, primary bile acid biosynthesis, glycerophospholipid metabolism, fatty acid degradation, biosynthesis of unsaturated fatty acids and fatty acid elongation (**Figure 4D**).

To validate the RNA-Seq data, four DEGs were selected for qPCR analysis. These genes included *lpin1a, alox5, abhd10* and *kif25*. As shown in **Figure 4E**, the genes showed higher expression level in the *GH* transgenic common carp liver determined by qPCR, consistent with the RNA-seq analysis results.

### Growth and feed utilization

**Figure 5:**
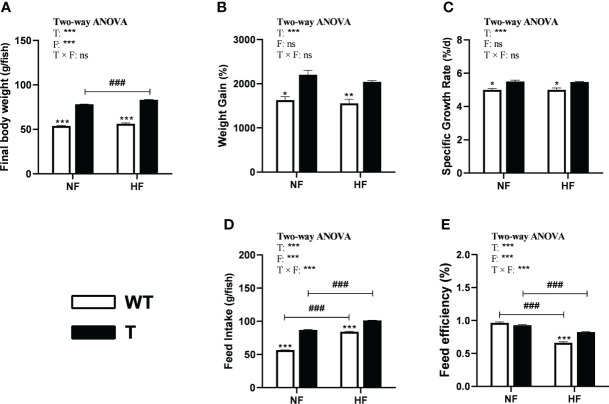
Effects of GH overexpression on growth performance in common carp fed different lipid levels diets. NF diet, 6% lipid; HF diet, 12% lipid. **(A–E)** Final body weight, Weight gain, Specific growth rate, Feed intake, and Feed efficiency of wild-type (WT) and GH transgenic **(T)** common carp. *, ** and *** mean significant differences (*P* < 0.05), (*P <* 0.01) and (*P* < 0.001) at same dietary lipid content for different strains. ### means significant difference (*P* < 0.001) between different lipid content for one strain. In two-way ANOVA analysis, T, GH transgenic. F, fat, ***P < 0.001. ns, no significance.

In both the NF and HF groups, the WG and SRG of *GH* transgenic common carp were significantly higher than those of WT common carp, which did not alter with change in dietary lipid levels (**Figure 5B, C**). In the NF and HF groups, the final body weight and feed intake amount of *GH* transgenic common carp increased significantly compared to WT common carp. The final body weight and feed intake of *GH* transgenic common carp, and feed intake of WT common carp significantly increased when dietary lipid level increased (**Figure 5A, D**). In the NF group, there was no difference in the FE between *GH* transgenic and WT common carp. when dietary lipid level increased, the FE of both strains decreased significantly. Moreover, in the HF group, the FE of *GH* transgenic common carp was significantly higher than that of the WT common carp (**Figure 5E** and **Supplementary Table 5**).

## Discussion

In this study, we found that *GH* transgenic and WT common carp mobilized glycogen firstly and then lipid for energy supply. In addition, *GH* overexpression primarily enhanced lipid catabolism in liver of the common carp. Under HF nutrition condition, common carp maintained high growth performance by intaking more feed. Compared with WT carp, *GH* transgenic common carp were more adaptable to the unfavorable nutritional environment of high-level dietary fat.

Different fish species had different strategies in utilizing their energy reserves such as glycogen, lipid, and protein during starvation (46). Some fish firstly used glycogen for energy in starvation (47), such as the gibel carp (*Carassius auratus gibelio* var. CAS III) (48). Atlantic cod (*Gadus morhua* L.) utilized lipid as their primary fuel source (49), and goldfish (*Carassius auratus*) initially mobilized muscle protein (46). A previous study in our laboratory has shown that there was no significant difference of basal metabolic traits associated with standard metabolic rate and energy change during starvation between *GH* transgenic and WT common carp (50). However, for *GH* transgenic and WT common carp, the order of utilizing glycogen, lipid, and protein during short-term fasting has not yet been reported. Thus, before the growth experiment, in which the common carp was fed and then in short time starvation, we need to ascertain if the *GH* transgenic and WT common carp utilize energy reserves in the same order. In the present study, we found that under short-term starvation condition, *GH* transgenic and WT common carp mobilized glycogen, lipid and protein in the same order, glycogen was mobilized firstly, and lipid was mostly utilized after glucose catabolism.

We found that the lipid content in the serum, liver and whole body of the *GH* transgenic common carp was significantly reduced, and the abilities of hepatic lipolysis and fatty acid β-oxidation were enhanced, which indicated that the *GH* transgene enhanced the ability of lipid catabolism in the liver of common carp. GH could improve energy supply and prevent excessive lipid deposition in tissues through lipolysis in fish (51). Previous studies showed that the lipid content of the *GH* transgenic common carp was lower than that of WT common carp (10), and the lipid content of the whole body decreased in *GH* transgenic Atlantic salmon (12, 52). The liver was thought to be the major organ for lipid storage in fish (53). In our research, the activities of lipolytic enzymes HSL and ATGL and the expression level of *hsl* significantly increased in the liver of *GH* transgenic common carp. Studies in mammals also suggested that GH promoted lipolysis by increasing HSL activity (54, 55). Our previous study found that the expression level of the lipolysis-promoting hormone *sla* was significantly upregulated in *GH* transgenic common carp (31). These results showed that lipolysis was enhanced in the liver of *GH* transgenic common carp.

It was found that the number of mitochondria increased and the expression level of genes related to fatty acid β-oxidation (*cpt-1a* and *cpt-1b*) were upregulated in the liver of *GH* transgenic common carp. In addition, Guo etal. (32) found that the expression level of fatty acid oxidation-related genes in the liver of *GH* transgenic common carp was higher than that in WT carp. CPT1 is a key enzyme in fatty acid β-oxidation that transfers fatty acids from the cytoplasm to mitochondria to generate energy by β-oxidation (56). These results indicated that fatty acid β-oxidation was enhanced in the liver of *GH* transgenic common carp.

In addition, transcriptome analysis revealed that the expression level of genes related to lipolysis (*lpin1a* and *alox5*) and the expression level of genes related to the synthesis and degradation of ketone bodies (intermediate products of fatty acid β-oxidation pathway) (*abhd10* and *kif25*) was significantly upregulated in the liver of *GH* transgenic common carp. For common carp, the age of 6 months is one of the fastest-growth periods for outdoor culture in local pond. From the view of aquaculture, our data was supposed to be helpful to the future industrialization and feed development of *GH* transgenic common carp, therefore 6-month-old fish was used for transcriptome analysis. Indeed, the analysis result of the lipid metabolism of 2-month-old fish was consistent with that of 6-month-old fish, indicating that liver lipolysis and fatty acid β-oxidation were enhanced in *GH* transgenic common carp, even at different ages.

However, Leggatt et al., (28) found that the activity of lipid synthesis enzymes (α-Glycerophosphate dehydrogenase, α-GPDH) was significantly enhanced in the liver of the *GH* transgenic coho salmon. Rise etal. (26) found that the expression level of lipid synthase-related genes was upregulated in the liver of *GH* transgenic coho salmon. Hill et al. (57) found that no significant differences in the activity of hydroxyacyl-CoA dehydrogenase (HAD) related to fatty acid oxidation between *GH* transgenic and WT coho salmon. Dietary protein and lipid contribute to the energy supply in fish. The protein (33.1%) and fat (6.7%) content of the diet in this study was lower than the optimal protein content (approximately 43%) and fat content (approximately 16%) for freshwater stages of coho salmon (28). Studies have reported that different dietary protein and fat level could alter the lipid metabolism of fish (58, 59). This might explain the difference in lipid metabolism between *GH* transgenic common carp and coho salmon. It also suggested the complexity of the effect of GH on lipid metabolism in fish.

Previous studies have shown that increasing dietary lipid level could improve the growth performance of fish (60), but excessive dietary lipid level would also lead to the decreased growth performance of fish (61). Since common carp is an important aquatic species, we assessed growth performance between *GH* transgenic and WT common carp fed with different lipid level diets. It might be helpful to develop new aquafeed adapted to growth characteristics of *GH* transgenic common carp. This study found that the final body weight and feed intake significantly increased, and the WG and SGR did not change, but the FE was significantly reduced in *GH* transgenic common carp in the HF group than in the NF group, as the same as in the comparison of WT common carp (expect for final body weight, which did not change) in the NF groups and HF groups. These data indicated that common carp maintained their growth performance by increasing feed intake. It is worth noting that in the HF group, the WG, SRG and FE of *GH* transgenic common carp were significantly higher than those in WT common carp. This might be due to the following reasons: First, the level of available lipid of *GH* transgenic common carp increased in the HF group. As mentioned above, *GH* transgenic and WT common carp mobilized glycogen, lipid and protein in the same order. In this study, the starch level was the same in different diet groups, therefore, the increased lipid level in the HF group made more lipid available to common carp. Second, as mentioned above, the present study found that *GH* transgene enhanced lipolysis and fatty acid β-oxidation in the liver of common carp. Moreover, we found that the expression level of genes related to lipolysis (*hsl* and *atgl*) and fatty acid β-oxidation (*cpt-1b* and *pparα*) in the liver of *GH* transgenic common carp was significantly higher than that in WT common carp when fed with a HF diet, which was consistent with the result when fed with a NF diet (data not shown). Previous studies have also reported that SGR and FE decreased in *GH* transgenic coho salmon fed a high-protein-high-fat diet compared to that in *GH* transgenic coho salmon fed a high-protein-low-fat diet, but SGR and FE substantially increased in *GH* transgenic coho salmon compared to that in WT coho salmon fed a high-protein-high-fat diet (62). These results indicated that *GH* transgenic common carp could utilize lipid more effectively under high lipid level dietary condition, which led to better growth performance and FE than WT common carp.

In conclusion, our study found that *GH* overexpression enhanced lipid catabolism and utilization in the liver of common carp by upregulating the lipolysis and fatty acid β-oxidation pathways. Meanwhile, *GH* transgene did not modify the basal metabolic traits of common carp. *GH* transgenic and WT common carp mobilized glycogen firstly and then lipid for energy supply under short-term starvation. These characteristics led to lower lipid content at normal dietary lipid level and better growth performance at high dietary lipid level for *GH* transgenic common carp than WT common carp. Also, *GH* transgenic common carp could be used as a model to study highly efficient lipid utilization in fish.

## Data availability statement

The datasets presented in this study can be found in online repositories. The names of the repository/repositories and accession number(s) can be found below: https://www.ncbi.nlm.nih.gov/, PRJNA849151.

## Ethics statement

All animal procedures were approved by Laboratory animal welfare and ethics committee, Institute of hydrobiology, Chinese Academy of Sciences.

## Author contributions

ZZ and WH contributed to the conception, supervision, andfunding acquisition of the study. YYW, JC and WH wrote the manuscript. YYW, RL, WG, YL, DH, SX, YPW and WH performed the experiments. YYW, XW, YS, BT, JC and WH did data analysis. All authors read and approved this manuscript.

## Funding

This study was supported by National Natural Science Foundation of China (31721005, 31672661).

## Acknowledgments

We would like to thank Mr. Xin Wang (Analytical and Testing Center, Institute of Hydrobiology, Chinese Academy of Sciences) for Micro-CT experimental support.

## Conflict of interest

The authors declare that the research was conducted in the absence of any commercial or financial relationships that could be construed as a potential conflict of interest.

## Publisher’s note

All claims expressed in this article are solely those of the authors and do not necessarily represent those of their affiliated organizations, or those of the publisher, the editors and the reviewers. Any product that may be evaluated in this article, or claim that may be made by its manufacturer, is not guaranteed or endorsed by the publisher.
