# Supplementary material for: Improved liver lipid catabolism and utilization in growth hormone transgenic common carp (Cyprinus carpio L.) through enhanced lipolytic and fatty acid β-oxidation pathways
Source: Front Endocrinol (Lausanne). 2022 Sep 12;13:982488. doi: 10.3389/fendo.2022.982488 (PMC9510774; doi:10.3389/fendo.2022.982488)
Supplement: Supplementary file 1 [file Table_1.docx]

**Formulation and proximate composition of experimental diets (% dry matter).**

| **Ingredients** | **NF** | **HF** |
| --- | --- | --- |
| **White fishmeal** | 10.00 | 10.00 |
| **Soybean meal** | 16.40 | 16.40 |
| **Casein** | 20.50 | 20.50 |
| **Corn starch** | 30.00 | 30.00 |
| **Fish oil** | 2.30 | 5.30 |
| **Soybean oil** | 2.30 | 5.30 |
| **Carboxymethyl cellulose sodium** | 3.00 | 3.00 |
| **Cellulose** | 10.00 | 4.00 |
| **Choline chloride** | 0.11 | 0.11 |
| **Vitamin premix^a^** | 0.39 | 0.39 |
| **Mineral premix^b^** | 5.00 | 5.00 |
| **Chemical composition(%）** |  |  |
| **Moisture** | 7.93 | 5.22 |
| **Crude protein** | 33.13 | 33.57 |
| **Crude lipid** | 6.68 | 12.68 |
| **Ash** | 6.49 | 6.74 |
| **Gross energy (Kj/g dry matter)** | 19.63 | 21.26 |

^a^ Vitamin premix (mg kg^−1^ diet): Thiamin, 20; Riboflavin, 20; Pyridoxine, 20; Cyanocobalamine, 0.02; Folic acid, 5; Calcium pantothenate, 50; Inositol, 100; Niacin, 100; Biotin, 0.1; Cellulose, 3412; Ascorbic acid, 100; Vitamin A, 11; Vitamin D, 2; Vitamin E, 50; Vitamin K, 10.

^b^ Mineral premix (mg kg^−1^ diet): NaCl, 500; MgSO_4_·7H_2_O, 8155.6; NaH_2_PO_4_·2H_2_O, 12,500.0; KH_2_PO_4_, 16,000.0; CaHPO_4_·2H_2_O, 7650.6; FeSO_4_·7H_2_O, 2286.2; C_6_H_10_CaO_6_·5H_2_O, 1750.0; ZnSO_4_·7H_2_O, 178.0; MnSO_4_·H_2_O, 61.4; CuSO_4_·5H_2_O, 15.5; CoSO_4_·7H_2_O, 0.91; KI, 1.5.
