# Supplementary material for: Improved liver lipid catabolism and utilization in growth hormone transgenic common carp (Cyprinus carpio L.) through enhanced lipolytic and fatty acid β-oxidation pathways
Source: Front Endocrinol (Lausanne). 2022 Sep 12;13:982488. doi: 10.3389/fendo.2022.982488 (PMC9510774; doi:10.3389/fendo.2022.982488)
Supplement: Supplementary file 2 [file Table_2.docx]

**Primer sequences used for the analysis of expression level of genes involved in metabolism by qPCR**

| **Gene name** | **Accession no.** | **Sequences (5′–3′)** |
| --- | --- | --- |
| ***srebp*** | KJ162572 | **F** CGTCTGCTTCACTTCACTACTC  **R** GGACCAGTCTTCATCCACAAA |
| ***fas*** | KY378913.1 | **F** CAGCGCCGCCACGACAACCT  **R** GGCCTCCGCTGGCACAGGAA |
| ***accα*** | XM_042723589.1 | **F** CCGGGCCGCATGCAGGAGAA  **R** TGGCCGTCCGTCAGCTCACTGTT |
| ***hsl*** | MF061228.2 | **F** GCCCTGGGCCAGTCAAAGTGC  **R** CCCTCGGGACGAGAAAGGGCCAA |
| ***atgl*** | KY906167.1 | **F** ACAGGATCACTCCAGCATCG  **R** CCTCCACCAGCGCTTTATGT |
| ***pparα*** | FJ849065.1 | **F** TGGCTAAGATGGTGGGCTCT  **R** CTGGTCATTTAAGTCCAGGTTTG |
| ***cpt-1a*** | KP262350.1 | **F** CGCGAGGGCCGGACGGAAAC  **R** TCGTGGTGCTCTCGTCCTCCA |
| ***cpt-1b*** | KJ816746.1 | **F** GCATAGACTCACCCTTCCTCAAA  **R** GAATGTCGATGAGATTGAGCTGC |
| ***hk1*** | XM_042736852.1 | **F** TGGCGCTACTGCAGGTCAGGT  **R** AGCCCGGCGGGAAACAGCTC |
| ***pk1*** | XM_042715494.1 | **F** AACGACGTGTGGGCCGAGGA  **R** TGGACGCCAGCCGGTCAGGA |
| ***pfk*** | XM_042764356.1 | **F** CACGTACAAGCTGTTAGCT  **R** TCGAAGCCATCATGGACGGT |
| ***g6p*** | AF427863.1 | **F** TGGTTGTTGCCGAGGCCTTCA  **R** TGGGCTTTCTCCAGGGTCCACAGC |
| ***fbp*** | AF427864.1 | **F** TGGTTCTCTCCACAGGCCAAGG  **R** GGGCGAACTTCCATCCTCTGGGA |
| ***pepck*** | KP250869.1 | **F** GGTGCCCTCTTTGACCTGCCCAA  **R** TCTGGCCTCCAGCGCCCTCA |
| ***gdh1α*** | XM_042736835.1 | **F** CCATCTCCTTATGTCTGTCCAGG  **R** GAACAATAGGTATGGCTCCACCA |
| ***asna*** | XM_042776724.1 | **F** GGAGCACATCGAGTCTGAGG  **R** CTCACAGCGGTTGGGGTAAT |
| ***mtor*** | FJ899680.1 | **F** ATCTACGGCAAGACGAGAGG  **R** GTTGGTGGAGAGTGGGATCA |
| ***pept1*** | JN896885.1 | **F** TCCAGGCTGGTTGGCTGTTG  **R** GCACGTATTCAGCCCACTGC |
| ***cytb*** | NC_001606.1 | **F** TTTGCCTACGCCATCCTACG  **R** ATGGGGCGGAATGTTAGTCC |
| ***β-actin*** | JQ619774.1 | **F** GATGATGAAATTGCCGCACTG  **R** ACCAACCATGACACCCTGATGT |

*srebp*, sterol regulatory element-binding proteins; *fas*, fatty acid synthase; *accα*, acetyl-CoA carboxylase α; *hsl*, hormone-sensitive lipase; *atgl*, adipose triglyceride lipase; *pparα*, peroxisome proliferators-activated receptor α; *cpt-1a*, carnitine palmitoyltransferase 1a; *cpt-1b*, carnitine palmitoyltransferase 1b; *hk1*, hexokinase 1; *pk1*, pyruvate kinase 1; *pfk*, phosphofructokinase; *g6p*, glucose-6-phosphatase; *fbp*, fructose bisphosphatase; *pepck*, phosphoenolpyruvate carboxykinase; *gdh1α*, glutamate dehydrogenase 1α; asna, asparagine synthetase; *mtor*, mammalian target of rapamycin; *pept1*, oligopeptide transporter 1; *ctyb*, mitochondrial cytochrome b
