# Supplementary material for: Improved liver lipid catabolism and utilization in growth hormone transgenic common carp (Cyprinus carpio L.) through enhanced lipolytic and fatty acid β-oxidation pathways
Source: Front Endocrinol (Lausanne). 2022 Sep 12;13:982488. doi: 10.3389/fendo.2022.982488 (PMC9510774; doi:10.3389/fendo.2022.982488)
Supplement: Supplementary file 3 [file Table_3.docx]

**Primer sequences used for validation of DEGs by qPCR.**

| **Gene name** | **Gene ID** | **Sequences (5′–3′)** |
| --- | --- | --- |
| ***lipin1a*** | carp1800842 | **F** CGTCTATGTGAGGTCGTGGATCATG  **R** AGGTGAACTGGCTGAAGGTGTCT |
| ***alox5*** | carpUN01671 | **F** TCTGCCTGACCGTGATTGTTCCT  **R** TCCTTCGCTGGTTGCTCTGTGA |
| ***abhd10*** | carp0600072 | **F** AAGAGTCCTGGTGTTGTGTTCCTG  **R** CATGTTCCAATGGTGCCTTCTGAA |
| ***kif25*** | carp0200622 | **F** AAGCAAGTCCAGCAACCGAGTG  **R** AGTCTGTTCCAGTTCAGCCTTCATT |
